# In Situ Atomic‐Scale Observation of Kinetic Pathways of Sublimation in Silver Nanoparticles

**DOI:** 10.1002/advs.201802131

**Published:** 2019-01-30

**Authors:** Junjie Li, Zhongchang Wang, Yunping Li, Francis Leonard Deepak

**Affiliations:** ^1^ Nanostructured Materials Group Department of Advanced Electron Microscopy Imaging and Spectroscopy International Iberian Nanotechnology Laboratory (INL) Avenida Mestre Jose Veiga Braga 4715‐330 Portugal; ^2^ Department of Quantum and Energy Materials International Iberian Nanotechnology Laboratory (INL) Avenida Mestre Jose Veiga Braga 4715‐330 Portugal; ^3^ Advanced Institute for Materials Research Tohoku University 2‐1‐1 Katahira Aoba‐ku Sendai 980‐8577 Japan; ^4^ State Key Lab for Powder Metallurgy Central South University Changsha 410083 China

**Keywords:** aberration‐corrected transmission electron microscopy, defects, in situ observation, kinetic process, phase transition, sublimation

## Abstract

Uncovering kinetics of sublimation atomically is critical to understanding both natural phenomena and advanced manufacturing technologies. Here, direct in situ atomic‐scale observations to understand the effects of size, surface, and defects in the sublimation process of supported silver nanoparticles upon heating within an aberration‐corrected transmission electron microscopy are conducted. Atomic‐scale evidence to sublimation and atomic rearrangement in small Ag nanoparticles during heating is provided, and it is demonstrated that the sublimation‐induced stable surfaces in the particles with a size smaller than ≈30 nm are {111} and {100} planes. The role of surface energy and defects in the uniform and nonuniform sublimation pathways at the atomic scale is also revealed, and it is found that the nanoparticles with low surface energy tend to undergo a uniform sublimation pathway, while those with high surface energy or five‐fold twin grain boundary proceed via a nonuniform sublimation pathway. Further dynamic analysis unravels a critical size of ≈8 nm for the transformation from linear to nonlinear sublimation rates in the two pathways. These findings demonstrate that the size, shape, and defects are of paramount importance for the sublimation dynamics in the first‐order phase transformation, helping to advance the general understanding of many technological applications.

## 1 Introduction

The process by which a crystal sublimates into a gas is a first‐order phase transition of considerable fundamental and practical importance in condensed matter physics, material science, and climate change, yet a detailed understanding of its relevant kinetic pathways is still evolving even for the model systems whose equilibrium configuration is known in advance.^1^, ^2^, ^3^, ^4^, ^5^, ^6^ For over a century, researchers have speculated on how ice sublimates and which key factors (e.g., particle size, morphology, defects) influence sublimation.^7^, ^8^, ^9^, ^10^, ^11^, ^12^, ^13^ Until a few decades ago, the equilibrium thermodynamic parameters, for example free energy, enthalpy, and vapor pressure, were investigated systematically in vaporization reactions.^14^, ^15^, ^16^ Transmission electron microscopy (TEM) in principle, enables us to observe dynamic process directly, yet to date many of the TEM studies on sublimation dynamics have been implemented on nanostructures at the nanometer scale, such as nanowires, nanorods, and colloidal nanocrystals.^12^, ^17^, ^18^, ^19^, ^20^, ^21^ Observing sublimation at the atomic scale has been mostly performed recently on 2D layered materials owing to their suitable thickness as a TEM sample in order to exclude the projection issues in electron microscopy.^22^, ^23^, ^24^

In case of metals, silver (Ag) nanocrystals with a face‐centered cubic (FCC) structure show a low sublimation temperature.^25^ As such, Ag represents an ideal model material to track heating‐induced sublimation procedure inside an electron microscope. For example, Mori and co‐workers reported in situ high‐resolution electron microscopy (HREM) observations of sublimation of nanometer‐sized Ag particles and found no occurrence of nanoparticle melting during sublimation by using a conventional electron microscope.^26^ Recently, direct observations of the sublimation of Ag nanoparticles were carried out at the nanoscale under heating in the transmission electron microscope, and a particle size‐dependent sublimation behavior was reported.^27^ Moreover, in situ observation of sublimation‐induced shape evolution of carbon covered silver cubes has also been conducted at the nanoscale in TEM.^21^, ^28^ Nevertheless, due to the limit of spatial resolution of a conventional TEM, many critical details such as structural evolution, dynamic processes, sublimation mechanisms, and role of defects during sublimation remain unclear as well as not very well established at the atomic scale.^18^ With the development of aberration‐corrected TEM (AC‐TEM) and advanced heating stage, in situ dynamic observations at atomic resolution can be achieved even at a low voltage, allowing us to directly resolve extremely small nanostructures that are not clearly visible by a conventional TEM.^29^, ^30^, ^31^

Here, we report the in situ atomic‐scale observation of the sublimation dynamics of Ag nanoparticles with a size smaller than ≈30 nm under heating conditions using an advanced heating holder within an AC‐TEM. The Ag nanoparticles are fabricated directly on the TEM heating chips by plasma irradiation in vacuum system to avoid unwanted solvent effects, and both plane view and cross‐sectional view of the sublimation dynamics are implemented at the atomic scale. We find surprisingly that the sublimation and atomic rearrangement coexist in small Ag nanoparticles during heating and reveal that the sublimation‐induced stable surfaces are the low‐energy {111} and {100} planes for the Ag nanocrystal with a size smaller than ≈30 nm. In addition to the size‐dependent sublimation behaviors, we also demonstrate that surface energy and defects are also of relevance in determining the sublimation pathways, that is, the Ag nanoparticles of a low surface energy show a uniform sublimation pathway, while those of a high energy or a five‐fold twin grain boundary undergo nonuniform sublimation pathways.

## 2 Results and Discussion

To avoid unwanted solvents, the Ag nanoparticles are directly fabricated by irradiating the Ag_2_WO_4_ nanorods by plasma onto a Si_3_N_4_ support in a vacuum system for ≈4 min. The Ag_2_WO_4_ nanorods are synthesized by a wet chemical route.^32^ X‐ray diffraction (XRD) analysis and TEM imaging (Figures S1 and S2, Supporting Information) show a successful preparation of Ag_2_WO_4_ nanorods with an orthorhombic structure (JCPDS Card No. 34‐0061). In situ heating experiments are conducted in an AC‐TEM equipped with a NanoEx‐i/v‐Single‐Tilt TEM heating holder, which allows a very rapid heating and cooling rate and possesses negligible thermal drift. To minimize the electron beam induced effects and to accurately measure sublimation temperature, an electron dose rate of ≈8.0 × 10^3^ e Å^−2^ s^−1^ and a low heating/cooling rate of ≈0.2 °C s^−1^ were adapted. It is known that Ag tends to segregate out of Ag_2_WO_4_ nanorods under irradiation to form Ag nanoparticles.^33^, ^34^

**Figure 1:**
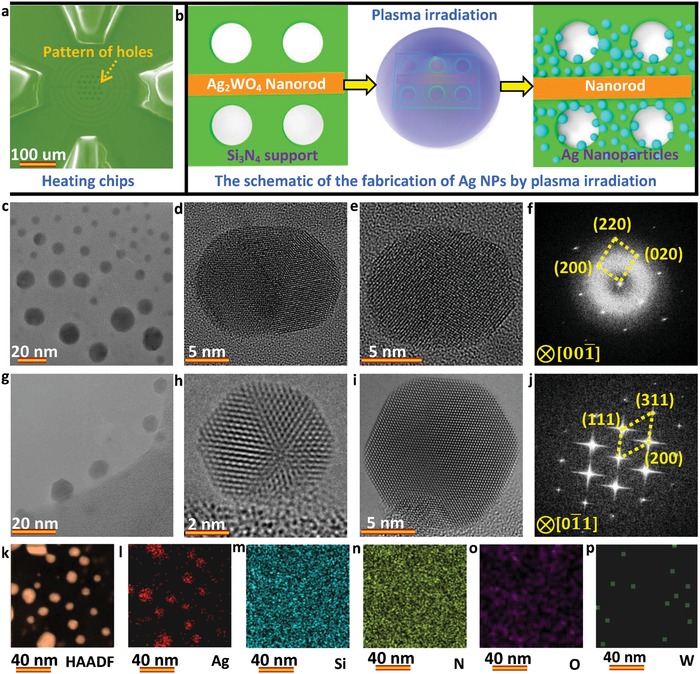
Fabrication of Ag nanoparticles on the heating chips through a plasma irradiation route. a) SEM image showing the pattern of holes in the Si_3_N_4_ membrane of the heating chips. b) Schematic illustration of the direct fabrication of Ag nanoparticles on the chips by plasma irradiation of the Ag_2_WO_4_ nanorods. c,g) Low‐magnification TEM images showing the morphology of the obtained Ag nanoparticles on the support. d,e,h,i) HRTEM images revealing the cubic structure for the formed Ag nanoparticles. f,j) Corresponding FFT analyses in (e) and (i). k) High angle annular dark field scanning transmission electron microscope image and l–p) the corresponding EDS mapping confirming the chemical composition of the formed nanoparticles.

**Figure** **1**b illustrates the fabrication of Ag nanoparticles on a heating chip (Figure 1a), and Figure 1c–j shows TEM images and fast Fourier transformation (FFT) patterns for the formed Ag nanocrystals. The high‐resolution TEM (HRTEM) images (Figure 1d,e,h,i) confirm the formed Ag nanoparticles with a cubic structure (JCPDS Card No. 04‐0783). By carrying out statistical analysis (Figure S3, Supporting Information), the size distribution of the formed nanoparticles was found to be in the ≈3–25 nm range, and dependent on the irradiation time. Interestingly, there are some twin defects in the formed Ag nanoparticles (Figure 1d,h), offering possibilities to directly probe the effect of defects on the sublimation process. Further energy‐dispersive X‐ray spectroscopy (EDS) mapping (Figure 1k–p) and spectrum (Figure S4, Supporting Information) confirm the chemical composition of the formed Ag nanoparticles, ruling out possible presence of any other elements. To select a suitable sublimation temperature for the kinetic observations, the size‐dependent sublimation process is studied initially, as shown in Figures S5 and S8 (Supporting Information). The plane‐view imaging of Ag nanoparticles shows a size‐dependent initiating temperature for sublimation, and a temperature of 650 °C is adapted for following sublimation experiments owing to the proper sublimation rate. Meanwhile, two types of typical sublimation pathways are observed (Figure S5, Supporting Information): a uniform sublimation pathway (marked by a red box in Figure S5a–n, Supporting Information, and enlarged in Figure S6, Supporting Information) and a split nonuniform sublimation pathway (marked by a yellow box in Figure S5a–l, Supporting Information, and enlarged in Figure S7, Supporting Information). The sublimated atoms near to the substrate can be recaptured by the substrate and migrate on the substrate due to the chemical or physical adsorption. The outmost layers of the nanocrystals in the enlarged images (Figures S6c,h,i and S7b,e–g, Supporting Information) are believed to be related to the residual interfacial Ag atoms, which are adsorbed by the silicon nitride substrate seen during under or over focus imaging.

**Figure 2:**
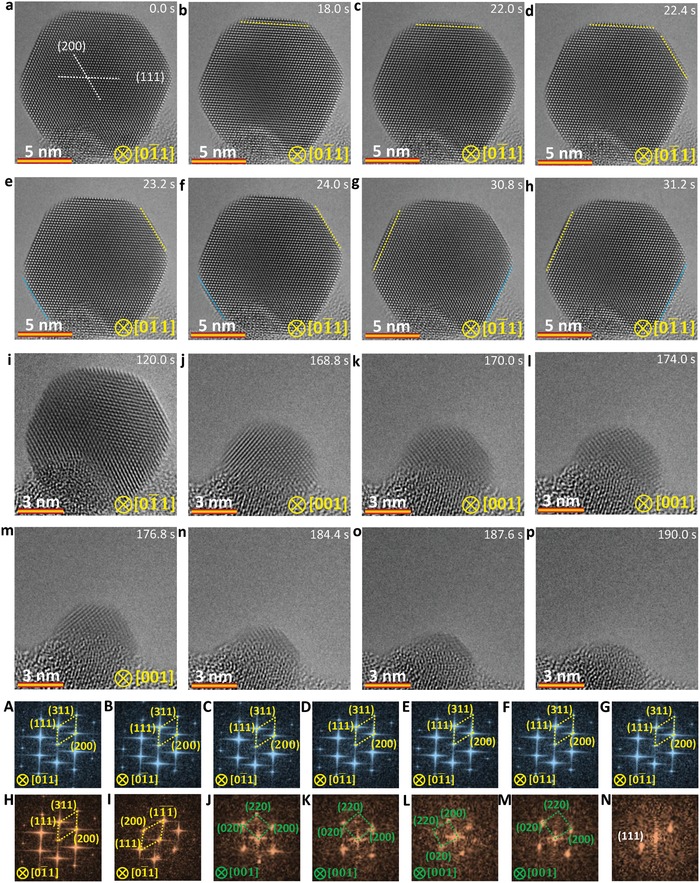
Sequential HRTEM images showing the dynamics of the uniform sublimation pathway for an Ag nanocrystal with a symmetrical structure. a–h) Sequential HRTEM images showing detailed layer‐by‐layer sublimation mechanism (indicated by yellow dashed lines in (a)–(h)) in the nanocrystal. The blue dashed lines in (e)–(h) indicate the accompanied atomic rearrangement during the sublimation. i–p) Sequential HRTEM images showing the accelerated sublimation and atomic rearrangement when the size of the nanocrystal is smaller than about 8 nm. a–n) Corresponding FFT analyses confirming the particle rotation during sublimation. The electron dose rate is 8.0 × 10^3^ e Å^−2^ s^−1^.

To gain insights into the two types of sublimation pathways at the atomic scale and to eliminate the effect of support on the image contrast, the nanoparticles in Figure 1g–i (the cross‐section view) are selected for kinetic observation of sublimation at 650 °C. **Figure** **2**a–p shows the kinetic pathway of the uniform sublimation of an Ag nanoparticle with a size of ≈15 nm at 650 °C viewed along [0$\bar{1}$1] direction (see also Video S1, Supporting Information). Based on the time‐sequential HRTEM images in both cross‐section view (Figure 2) and plane view (Figure S9, Supporting Information), one can see that the sublimation‐induced stable surfaces in the Ag nanocrystal are {111} and {100} planes, consistent with the low‐energy surfaces in face‐centered cubic noble metals, γ{111} < γ{100} < γ{110},^35^, ^36^ yet differs from the reported {110} plane for carbon‐covered Ag cubes with a size larger than 50 nm.^21^, ^27^ Figure S10 (Supporting Information) gives a different example of uniform sublimation in a symmetric nanocrystal with truncated structure at 600 °C, where the Ag nanocrystals of ≈15 nm also show a stable {111} surface during sublimation. These imply that the stable plane induced by sublimation is mainly affected by size and morphology of the nanocrystal. In Figure 2b–h, one can identify a symmetric layer‐by‐layer sublimation mode (marked by yellow dashed lines) so as to retain a stable height‐to‐width ratio. It is worthy of noting that atomic rearrangement is also observed during the layer‐by‐layer sublimation (indicated by blue dashed line in Figure 2e–h). The symmetrical sublimation and the associated atomic rearrangement are driven by surface energy minimization.^37^, ^38^, ^39^ When the size of Ag nanoparticles is smaller than ≈8 nm, both atomic rearrangement and sublimation are accelerated (Figure 2j–p). The corresponding FFT analyses in Figure 2A–N confirm the particle rotation of the Ag nanoparticle from 〈110〉 to 〈001〉 directions when the size is smaller than 8 nm.

**Figure 3:**
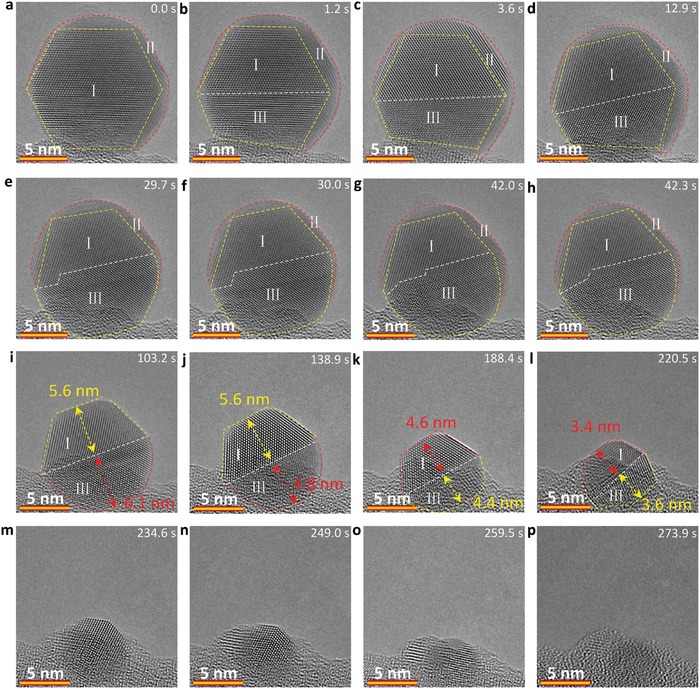
Sequential HRTEM images showing the dynamics of the nonuniform sublimation pathway for an Ag nanocrystal with an asymmetrical structure. a–i) Sequential HRTEM images showing the particle splitting during sublimation and preferential sublimation in a local region with a high surface energy. m–p) Sequential HRTEM images showing the accelerated sublimation and atomic rearrangement when the size of the nanocrystal is smaller than about 8 nm. The electron dose rate is 8.0 × 10^3^ e Å^−2^ s^−1^.

To uncover the kinetic pathway of nonuniform sublimation, an Ag nanocrystal of ≈15 nm with an asymmetric shape is heated from room temperature to 650 °C at a rate of 0.2 °C s^−1^. In contrast to the uniform sublimation pathway in the energy‐stable symmetrical nanocrystal (Figure 2), this Ag nanocrystal with asymmetrical structure takes a nonuniform pathway, which constitutes nanoparticle splitting during sublimation, as seen from the sequential HRTEM images viewed from [0$\bar{1}$1] direction in **Figure** **3** (see also Video S2, Supporting Information). In Figure 3a, one can identify a regular octagon‐shaped region that is composed by perfect region (far from support) and stacking faults region (close to support), and a region II between the yellow octagon and red line that shows different atomic arrangement. The region I is then split into region I and region III by a (111) twin plane (Figure 3b,c). Meanwhile, the region II shows a preferential sublimation in the following steps and the regions I and III show a size shrinkage due to atomic rearrangement (Figure 3b–h). When the size of the Ag nanoparticles is smaller than ≈13 nm, the region II vanishes (Figure 3i), while the region III shows preferential sublimation compared to region I (Figure 3i–j). To clearly show the preferential sublimation at this stage, we show more sequential images and indicate the shape evolution using yellow and red curves in Figure S11 (Supporting Information). Subsequently, the region I shows a preferential sublimation (Figure 3k–l). The preferential sublimation tends to take place in the bigger region during this stage (confirmed by measuring the layers of the lattice and the size of the total particle), which explains the fluctuation of preferential sublimation between regions I and III in Figure 3i–l. It is noteworthy that there is the atomic migration and rearrangement in the surface, but no obvious migration of twin boundary observed during this process. When the size becomes smaller than ≈8 nm, the nanoparticles show a uniform sublimation pathway, which is accompanied with a fast atomic rearrangement. During the whole process, the sublimation‐induced stable surface is the {111} plane. The nonuniform sublimation pathway undergoes nanoparticle spilt, rearrangement, and preferential sublimation in a local region, and finally uniform sublimation. Such a particle rotation in Figure 3 is further confirmed by the corresponding FFT analyses (Figure S12, Supporting Information). We further conduct over five individual sets of experiments and confirmed these observations. Figure S13 (Supporting Information) shows yet another example for the preferential sublimation in the top region (labeled as region I in Figure S13, Supporting Information), for which the sublimation‐induced stable surface is {111} plane.

To gain insights into the impact of shape of Ag nanoparticles on the sublimation pathways, we adopt an analytical model to calculate the energetics of sublimation for the Ag nanoparticles that undergo uniform sublimation and compare the energy to that required for sublimation via a local region preferring nonuniform sublimation pathway as proposed by Paulo et al.^27^ In their model, the change in energy during sublimation involves total surface energy of a nanoparticle, volume free energy of the sublimated vapor, and volume free energy of the solid nanoparticles. For the near‐spherical nanoparticles, total change in energy upon uniform sublimation can be expressed as^27^

(1) $$\Delta E_{\mathrm{uni}}\;=\;4\pi \gamma_{s}\left(r_{2}^{2}\;-\;r_{1}^{2}\right)\;+\;4/3\pi E_{\mathrm{sub}}\left(r_{2}^{3}\;-\;r_{1}^{3}\right)$$

where *r*_2_ = [*r*_1_^3^ − ¾ (*r*_1_ x^2^ − *x* ^3^/(4π))]^1/3^, *r*_1_ is the radius of the nanoparticles, γ_s_ is the surface energy per unit area, and *E*_sub_ is the heat of sublimation.

In case of the nonuniform sublimation, the change in total energy upon nonuniform sublimation can be expressed as

(2) $$\Delta E_{\mathrm{non}}\;=\;\pi \gamma_{s111}\left(2r_{1}x\;-\;x^{2}\right)\;-\;2\pi \gamma_{s}r_{1}x\;+\;\pi E_{\mathrm{sub}}\left(r_{2}^{3}/3\;-\;r_{1}x^{2}\right)$$

where γ_s111_ is the surface energy of the newly formed low‐energy {111} plane.

For a nanoparticle, the difference in change in energy for both the sublimation pathways can be expressed as

(3) $$\Delta E=\Delta E_{\mathrm{uni}}-\Delta E_{\mathrm{non}}$$

If the energy difference Δ*E* is negative, then

(4) $$\Delta E_{\mathrm{uni}}<\Delta E_{\mathrm{non}}$$

Since Δ*E*_uni_ and Δ*E*_non_ are both negative, the magnitude of the change in energy for the uniform sublimation is higher than that for the nonuniform one. Therefore, when Δ*E<*0, the uniform sublimation pathway is energetically favored, while the nonuniform sublimation pathway is energetically favored once the Δ*E* is positive.

**Figure 4:**
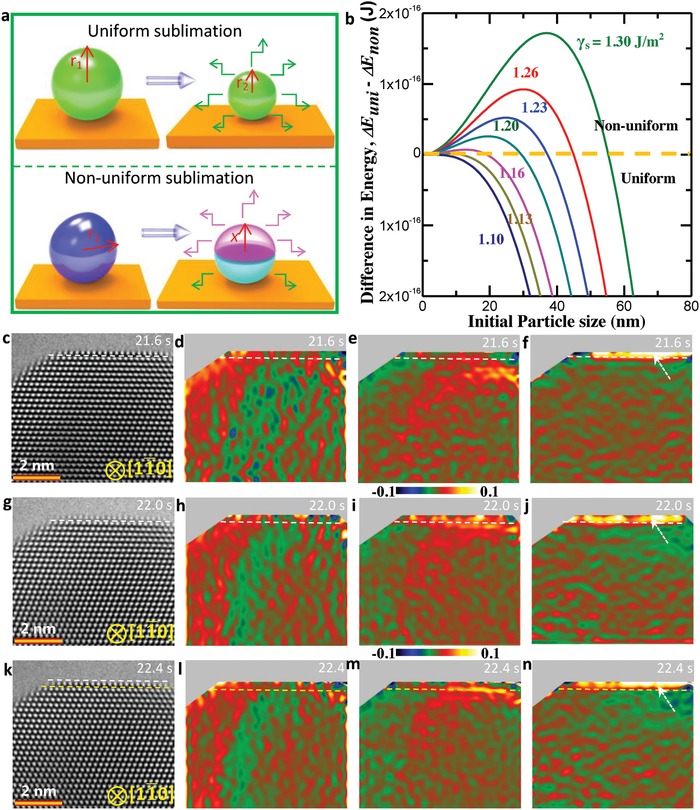
Surface energy effects on the uniform and nonuniform sublimation pathways. a) Schematic illustration of the uniform and nonuniform sublimation pathways for the Ag nanoparticles with a different morphology. b) Difference in the energy change for uniform and nonuniform sublimation as a function of particle size (*s*) in a series of surface energy density (γ_s_). c,g,k) Sequential HRTEM and strain distribution images: d,h,l) expansion ε_xx_, e,i,m) shear components ε_xy_, and f,j,n) contraction ε_yy_ showing the positive strain in the sublimated surface layer.

Based on Equations (1)–(3), the change in total energy in both the pathways is a function of nanoparticle size and surface energy. However, only the size effects on sublimation pathways were taken into account in previous studies. In our observation, the Ag nanoparticles possess a similar size, meaning that surface energy may play a role in the sublimation of nanoparticles. **Figure** **4**a sketches the uniform and nonuniform sublimation pathways and Figure 4b shows the difference in the Δ*E* for sublimation by uniform and nonuniform pathways as a function of the initial size *r*_1_ and surface energy obtained from Equations (1)–(3). The following values for the properties of Ag are adapted in the calculations: γ_s111_ = 0.62 J m^−2^, *E*_sub_ = 2.77 × 10^10^ J m^−3^, *x* = 0.9 *r*.^40^, ^41^ Based on the calculations, one can see that the size of the nanoparticles imposes an important influence on the sublimation pathways. Meanwhile, it is worthy of noting that the surface energy also plays an important role in deciding the sublimation pathways. The yellow dashed line in Figure 4b shows the critical value of surface energy density for uniform and nonuniform sublimation pathways, indicating that the nanoparticles with low surface energy tend to undergo a uniform sublimation pathway, while those with high surface energy favor the nonuniform sublimation pathway.

To shed more light on the layer‐by‐layer sublimation, we implement the sequential strain tensor analyses in the marked region (white box in Figure S14, Supporting Information) in Video S1 (Supporting Information), as shown in Figure 4c–n. The strain tensor maps of the corresponding HRTEM images are obtained using the geometrical phase analyses (GPA), where ε_xx_, ε_xy_, and ε_yy_ are expansion, shear components, and contraction of strain. Figure 4c,g,k shows the tailored time‐sequential HRTEM image in Video S1 (Supporting Information) at 21.6, 22.0, and 22.4 s, respectively, and Figure 4d,h,l shows the time‐sequential strain component of ε_xx_, Figure 4e,i,m in ε_xy_, and Figure 4f,j,n ε_yy_, respectively. The white lines mark the sublimated surface layer in Figure 4c–j. Based on the strain maps, one can notice that the topmost atomic layer on the surface shows the positive strain distribution to the center area in the strain component of ε_yy_ (marked by a white arrow in Figure 4f,j,n), indicative of the layer‐by‐layer sublimation mechanism for the Ag nanocrystal. Therefore, the surface atomic structure plays an important role in deciding the sublimation position and pathway because the surface structure and surface energy can affect the atomic rearrangement and migration.

**Figure 5:**
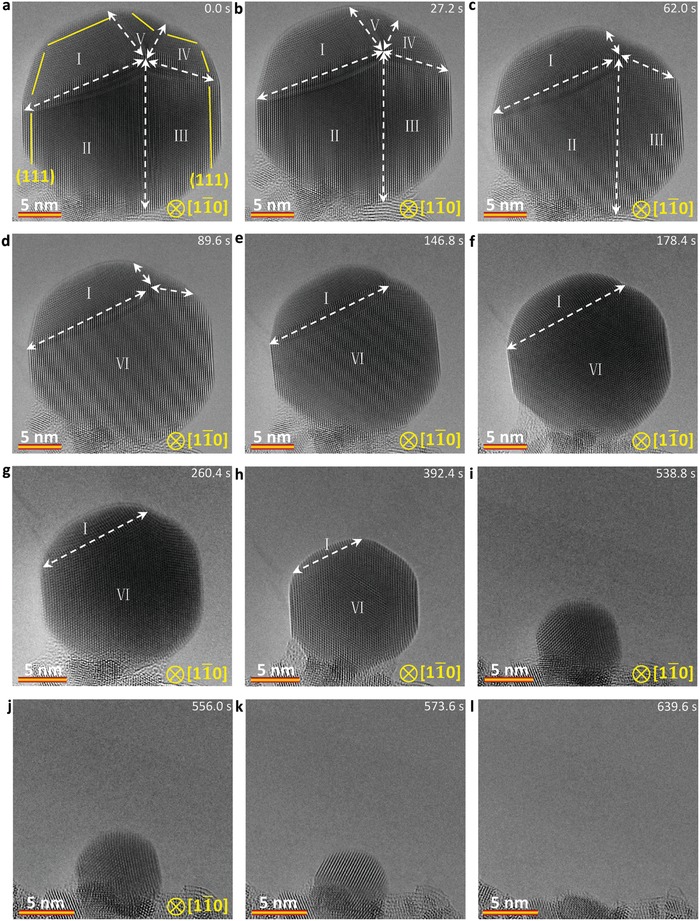
Sequential HRTEM images showing the dynamics of the defect structure induced nonuniform sublimation pathway for an Ag nanocrystal with a fivefold twin grain boundary. a–h) Sequential HRTEM images showing the preferential sublimation in region I, V, and IV. h–l) Sequential HRTEM images showing the accelerated sublimation and atomic rearrangement when the size of the nanocrystal is smaller than about 8 nm. The electron dose rate is 8.0 × 10^3^ e Å^−2^ s^−1^.

To uncover the effect of defects on sublimation, a series of in situ heating experiments of Ag nanocrystals with defects are carried out. **Figure** **5** shows a typical time‐sequential HRTEM images of sublimation of an Ag nanocrystal with a fivefold twin boundary at 650 °C (see also Video S3, Supporting Information). In Figure 5a, one can note the five‐fold twin boundary in the nanocrystal, which is also confirmed by a detailed FFT analysis (Figure S15 and Table S1, Supporting Information). The observed direction is close to [0$\bar{1}$1] Ag. The nanocrystal experiences a nonuniform sublimation pathway (Figure 5a‒i). One can further see that the nanocrystal shows a preferential sublimation in the regions I, IV, and V at the initial stage (Figure 5a‒h). Meanwhile, the atomic rearmaments give rise to the twin boundary between regions II and III, which finally moves out of the nanocrystal (Figure 5a‒d). When the size of nanoparticle is smaller than a critical size of about ≈8 nm, the atomic rearrangement in the small nanoparticle is enhanced at the high temperature. The particle can rearrange to an energy stable state, then the twins vanish, resulting in symmetric uniform sublimation (Figure 5i‒l). The viewing direction of the sublimated Ag nanocrystal is changed during the process due to the enhanced atomic rearrangement in the small nanoparticle at a high temperature (Figure S16, Supporting Information). In this nonuniform sublimation pathway, the stable grain boundaries divide the nanocrystal into five domains and show a preferential sublimation in some local ones. The preferential sublimation in the nanocrystal should be related to the increased surface and total energies by the five‐fold twin boundary. Although the increased surface energy is difficult to be quantitatively compared to the energy stable state without defects in the nanocrystal, the experimental observations here and the calculated results in Figure 4b provide insights into the possible reasons (change in the surface structure and increase of the surface energy) for the defects effected nonuniform sublimation mechanism. These results imply that defects can also have influence on the sublimation pathways.

**Figure 6:**
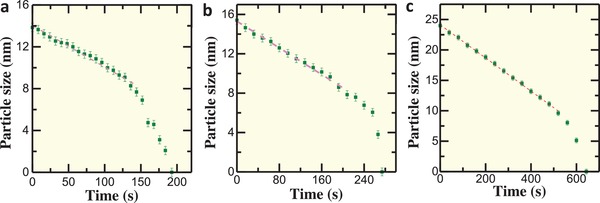
Statistical analysis of sublimation dynamics at 650 °C from Videos S1–S3 (Supporting Information). a) Statistical analysis of the particle size as a function of time for the uniform sublimation in Figure 2. b) Statistical analysis of the particle size as a function of time for the nonuniform sublimation in Figure 3. c) Statistical analysis of the particle size as a function of time for the nonuniform sublimation in Figure 5. The pink dashed lines represent the linear fitted results when the particle size is larger than ≈8 nm. The error bar is 0.4 nm.

To further probe the sublimation kinetics for both the uniform and nonuniform sublimation pathways in Figures 2, 3, and 5 and Figure S13 (Supporting Information) at 650 °C, we plot size of nanoparticle (*s* = (height + width)/2) as a function of sublimation time, as shown in **Figure** **6** and Figure S17 (Supporting Information). One can see that the observed Ag nanocrystals show a nearly constant sublimation rate until the size of nanoparticle is reduced below ≈8 nm, where the rate starts to increase significantly, then the nanocrystals show a quick sublimation in a nonlinear fashion. These results agree with the previous prediction of bulk thermodynamics based on the Kelvin equation and kinetic theory.^42^, ^43^ To investigate the nearly constant sublimation rate in the linear portion, we fit the experimental results using the size of nanoparticle of above 8 nm, as marked by pink dashed line in Figure 6. The slopes are calculated to be −0.0398, −0.0338, −0.027, and −0.03689 in Figure 6a–c and Figure S17 (Supporting Information), respectively. The results imply that the sublimation in Video S1 (Supporting Information) has a quicker sublimation rate than the ones in Figures 3 and 5 and Figure S13 (Supporting Information) at 650 °C.

## 3 Conclusion

The ability to conduct in situ atomic‐scale observation of kinetic pathways of sublimation in nanomaterials represents a significant step forward in understanding atomic mechanisms of the solid‐gas phase transitions. We conduct direct atomic imaging of the uniform and nonuniform sublimation pathways in solid‐to‐gas first‐order phase transformation and provide evidence that the sublimation‐induced stable surface in the Ag nanocrystal with a size smaller than ≈30 nm is the {111} and {100} planes rather than the {110} plane in large Ag particles. We identify atomic rearrangement during sublimation and reveal how size, surface, and defects affect the sublimation pathways and dynamics. The results show that the particles with a low surface energy tend to undergo a uniform sublimation pathway, while those with a high surface energy or fivefold twin grain boundary take the nonuniform sublimation pathway. Further dynamic analysis reveals a critical size of ≈8 nm for the transformation from linear to a nonlinear sublimation rates for the two pathways. The findings demonstrate that the solid‐to‐gas transformation of nanoparticles cannot be regarded solely as a size‐dependent phenomena but involves both size and surface energy (morphology), advancing our understanding of a range of technological applications, for example, purification, thin film deposition, nanoparticle growth, and patterning of microelectromechanical systems (MEMS).

## 4 Experimental Section

*Sample Preparation and Characterization*: Wet chemical method was used to synthesize the Ag_2_WO_4_ nanorods.^32^ In a typical synthesis process, 1.0 mmol tungstate sodium dihydrate (Na_2_WO_4_ • 2H_2_O, 99.95%) and 2.0 mmol silver nitrate (AgNO_3_, 99.8%) were dissolved separately in 50 mL deionized water. The solution of tungstate sodium dehydrate was transferred to a 200 mL glass flask and heated to 90 °C under quick stirring for 15 min. Subsequently, 50 mL solution of silver nitrate was poured into a hot glass flask, and a suspension of Ag_2_WO_4_ nanorods was rapidly formed, which was accompanied with temperature decrease to ≈70 °C. The resulting suspension was immersed in a beaker with 50 mL ice water for 10 min. Subsequently, the fine white powder at the bottom of the glass flask was washed with deionized water several times to remove the solvent, followed by drying at room temperature. Chemical composition of the obtained products was determined by powder XRD (X'Pert PRO diffractometer, PANalytical).

*TEM/Scanning TEM Observation and EDS Analysis*: The as‐obtained white powder samples were dispersed in ethanol by ultrasonication for 10 min, and then a drop of the suspension of Ag_2_WO_4_ nanorods was transferred onto the heating device. The heating stage utilized a disposable MEMS device, which could serve as a heating component by connecting the stage to an external power supply as well as to a specimen support grid. After the ethanol was evaporated naturally at room temperature, the Ag_2_WO_4_ nanorods loaded heating chip was placed into a heating specimen holder for subsequent irradiation experiments. Prior to the in situ heating experiments, Ag_2_WO_4_ nanorods were irradiated by plasma in a vacuum system for 2–4 min at room temperature to form dispersed Ag nanoparticles on the Si_3_N_4_ support. The formed Ag nanocrystal was confirmed by HRTEM and EDS spectrum. Subsequently, the Ag nanoparticles were used for the sublimation experiments in TEM. TEM imaging was performed at 200 kV using a double‐corrected Titan Themis electron microscope with a spatial resolution of ≈0.8 Å. Time‐sequential TEM images were acquired by Tecnai Imaging and Analysis (TIA) software.

*Strain Fields Analysis*: To gain insight into strain fields of the sublimated nanocrystals, GPA were implemented, which allowed quantitative characterization of 2D strain tensor for each atomic column in the field of view. Accuracy of GPA technique in the measurement of atomic strain/stress was verified in a wide range of material systems.^44^, ^45^ The obtained time‐sequential aberration‐corrected HRTEM images were used for the measurements.

## Conflict of Interest

The authors declare no conflict of interest.

## Supporting information

Supplementary

Supplementary

Supplementary

Supplementary
