# Supplementary material for: In Situ Atomic‐Scale Observation of Kinetic Pathways of Sublimation in Silver Nanoparticles
Source: Adv Sci (Weinh). 2019 Jan 30;6(8):1802131. doi: 10.1002/advs.201802131 (PMC6468973; doi:10.1002/advs.201802131)
Supplement: Supplementary file 1 — Supplementary [file ADVS-6-1802131-s002.pdf]

Copyright WILEY-VCH Verlag GmbH & Co. KGaA, 69469 Weinheim, Germany, 2019.

ADVANCED SCIENCE
Open Access

Supporting Information

for *Adv. Sci.,* DOI: 10.1002/advs.201802131

In Situ Atomic-Scale Observation of Kinetic Pathways of Sublimation in Silver Nanoparticles

*Junjie Li, Zhongchang Wang, Yunping Li, and Francis Leonard Deepak**

Supplementary Information

In-situ Atomic-Scale Observation of Kinetic Pathways of Sublimation in Silver Nanoparticles

Junjie Li, Zhongchang Wang, Yunping Li and Francis Leonard Deepak*

Dr. J. Li, Dr. Z. C. Wang, Dr. F. L. Deepak

Department of Advanced Electron Microscopy, Imaging and Spectroscopy

International Iberian Nanotechnology Laboratory (INL)

Avenida Mestre Jose Veiga, Braga 4715-330, Portugal

*Email: leonard.francis@inl.int

Dr. Z. C. Wang

Advanced Institute for Materials Research

Tohoku University

2-1-1 Katahira, Aoba-ku, Sendai 980-8577, Japan

Prof. Y. Li

State Key Lab for Powder Metallurgy

Central South University

Changsha 410083, China

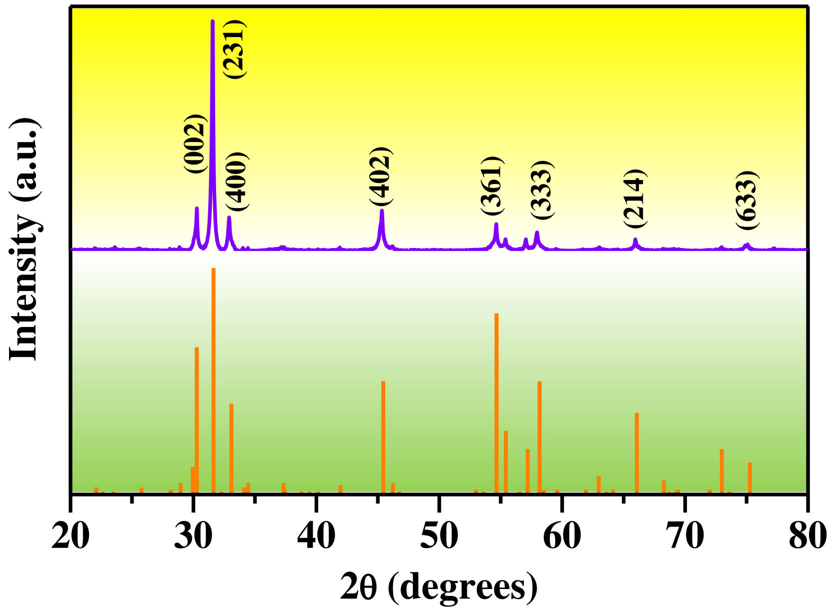

Supplementary Figure 1. XRD analysis. XRD results for the obtained Ag_2WO_4 nanorods and the corresponding standard pattern (JCPDS card no. 34-0061) revealing a successful preparation of pure Ag_2WO_4 with an orthorhombic structure.

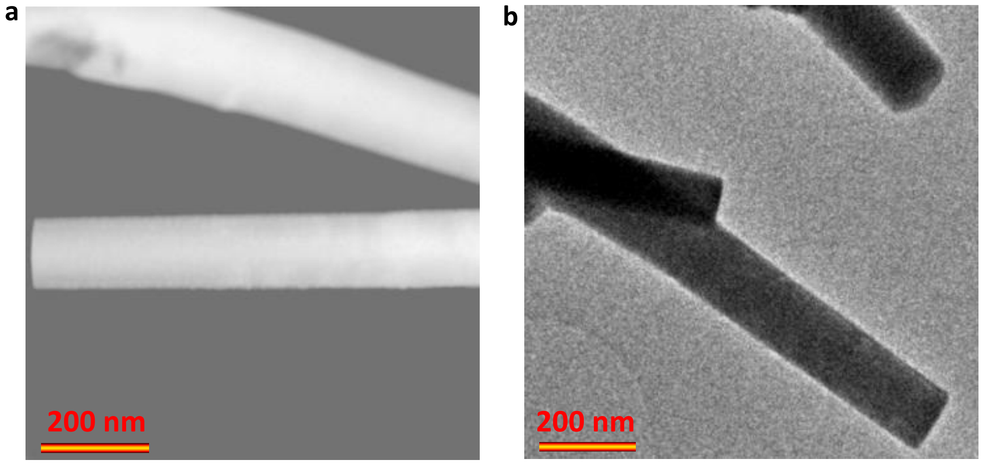

Supplementary Figure 2. Morphology of the obtained Ag_2WO_4 nanorods. Low magnification HAADF-STEM (**a**) and TEM (**b**) images for the obtained product.

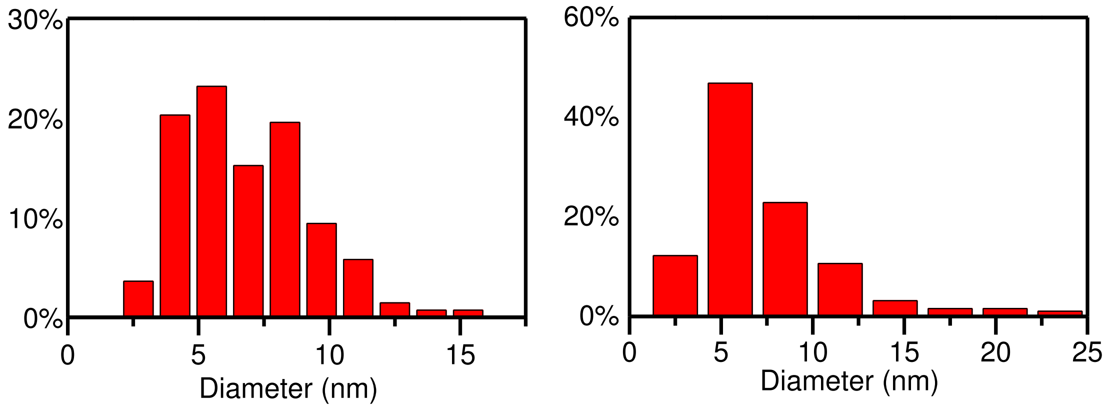

Supplementary Figure 3. The size distribution of formed Ag nanoparticles by plasma irradiation. **a,** The size distribution of the formed Ag nanoparticles by plasma irradiation for ~3 mins. **b,** The size distribution of the formed Ag nanoparticles by plasma irradiation for ~4 mins.

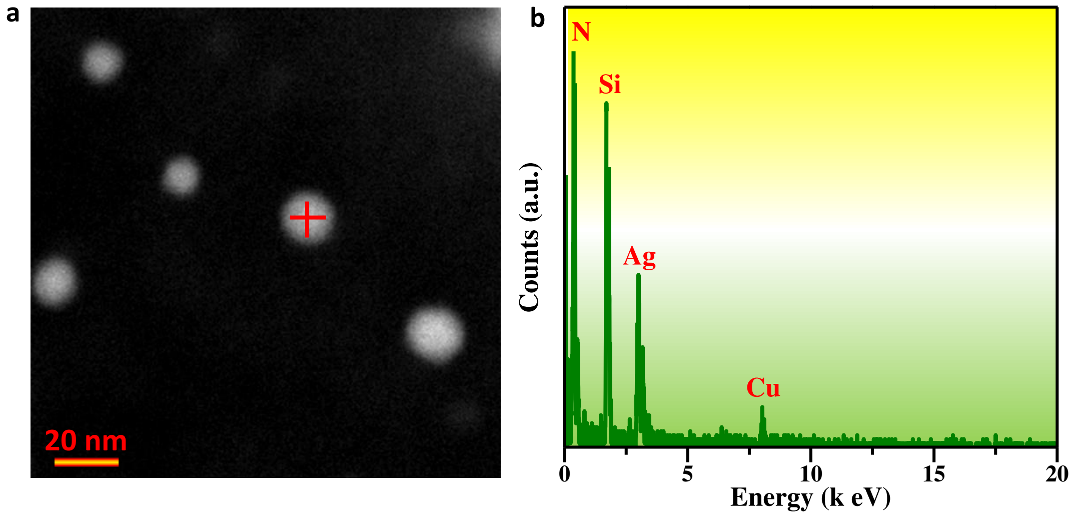

Supplementary Figure 4. HADDF-STEM image and EDS spectrum of Ag nanocrystal on Si_3N_4 support. **a,** HAADF STEM image of the formed Ag nanocrystals by plasma irradiating the Ag_2WO_4 nanorod for 3 mins onto the Si_3N_4 support. **b,** Corresponding energy-dispersive x-ray spectroscopy (EDS) spectrum of the Ag nanocrystal taken in the area marked by a red cross in **a**.

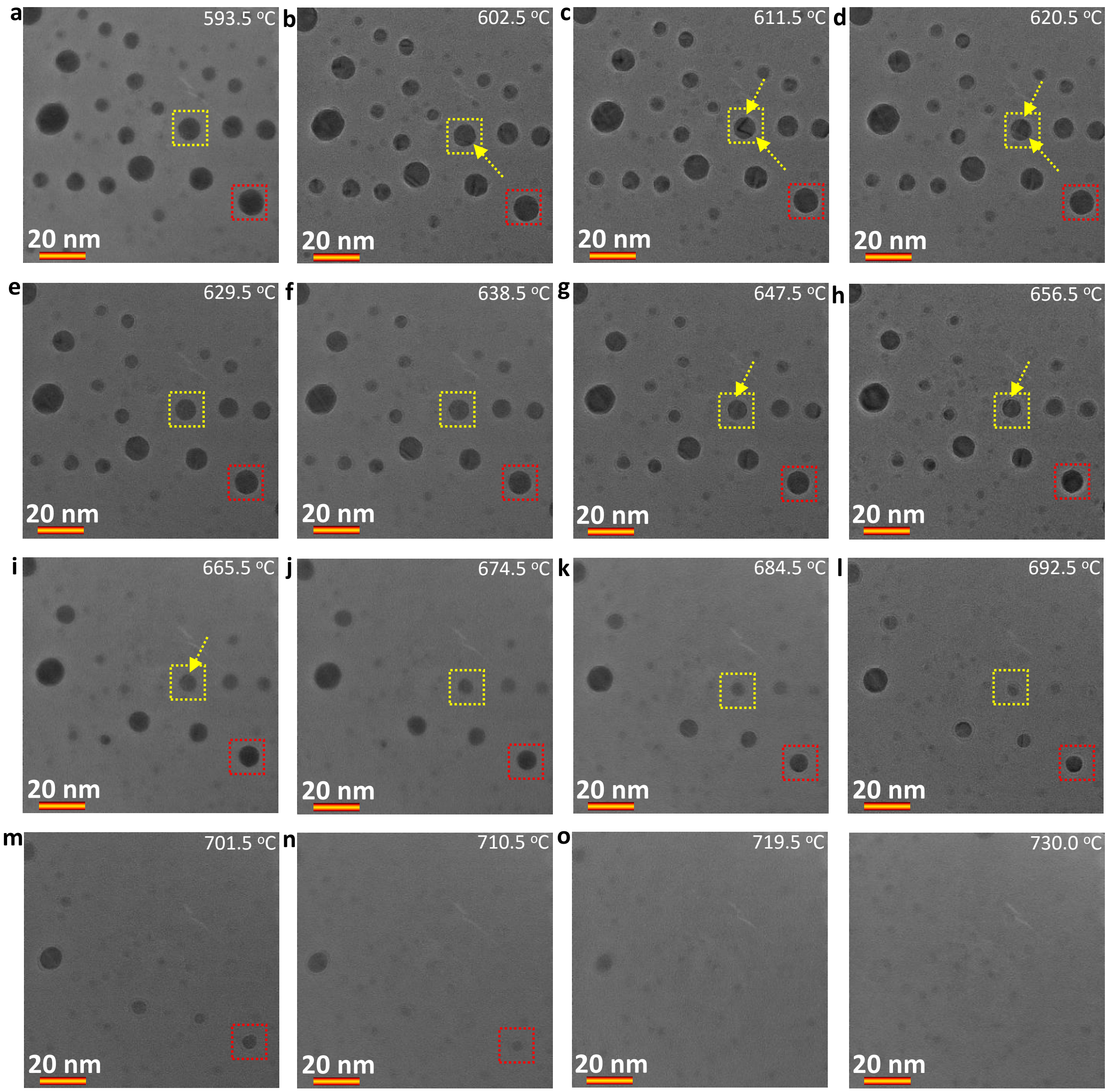

Supplementary Figure 5. Sequential high resolution TEM images ing size-dependent sublimation from ~593 ºC to ~730 ºC. The yellow arrows indicate the newly formed grain boundary during the non-uniform sublimation.

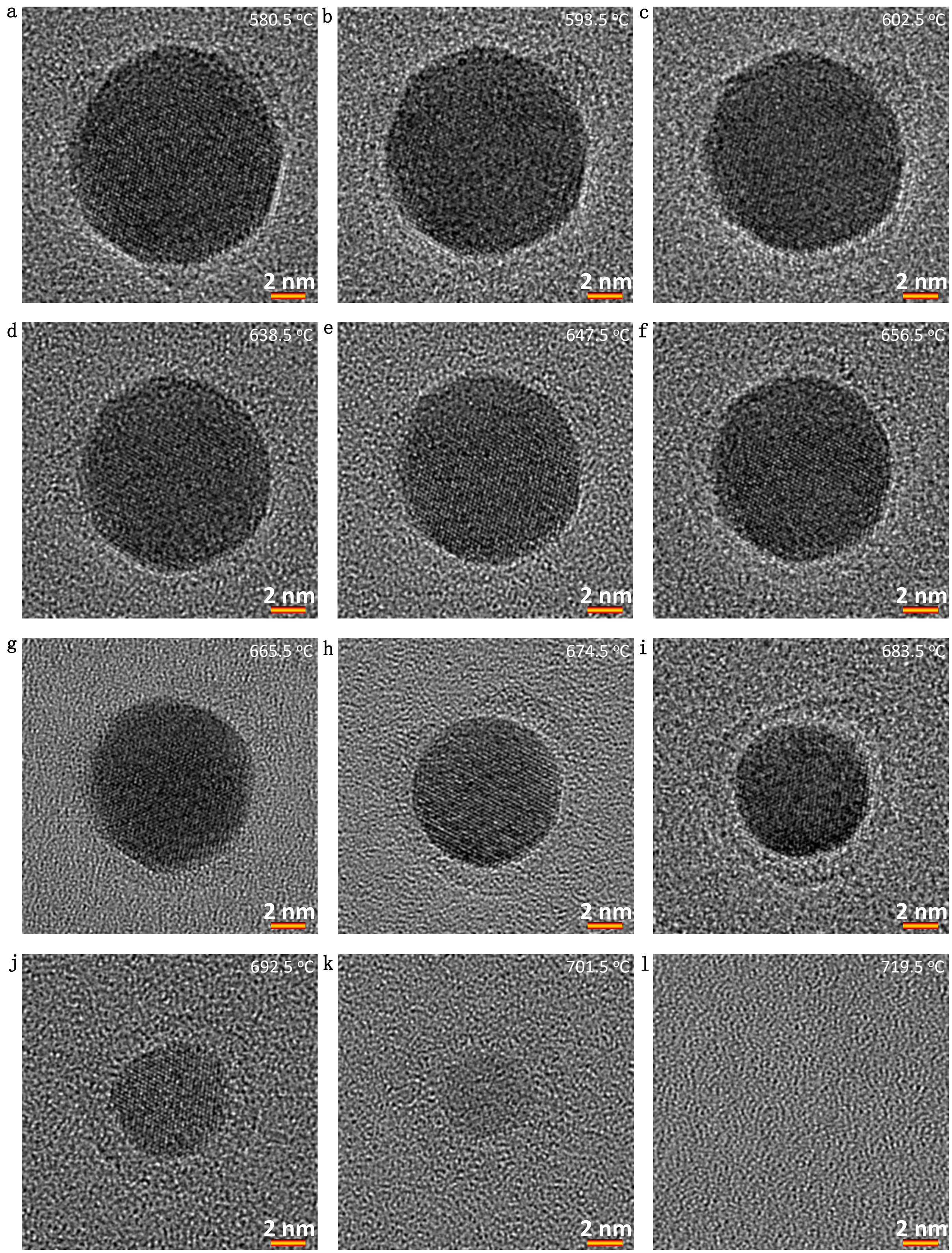

Supplementary Figure 6. The Enlarged sequential HRTEM images of the red box region in Figure S5 showing uniform sublimation.

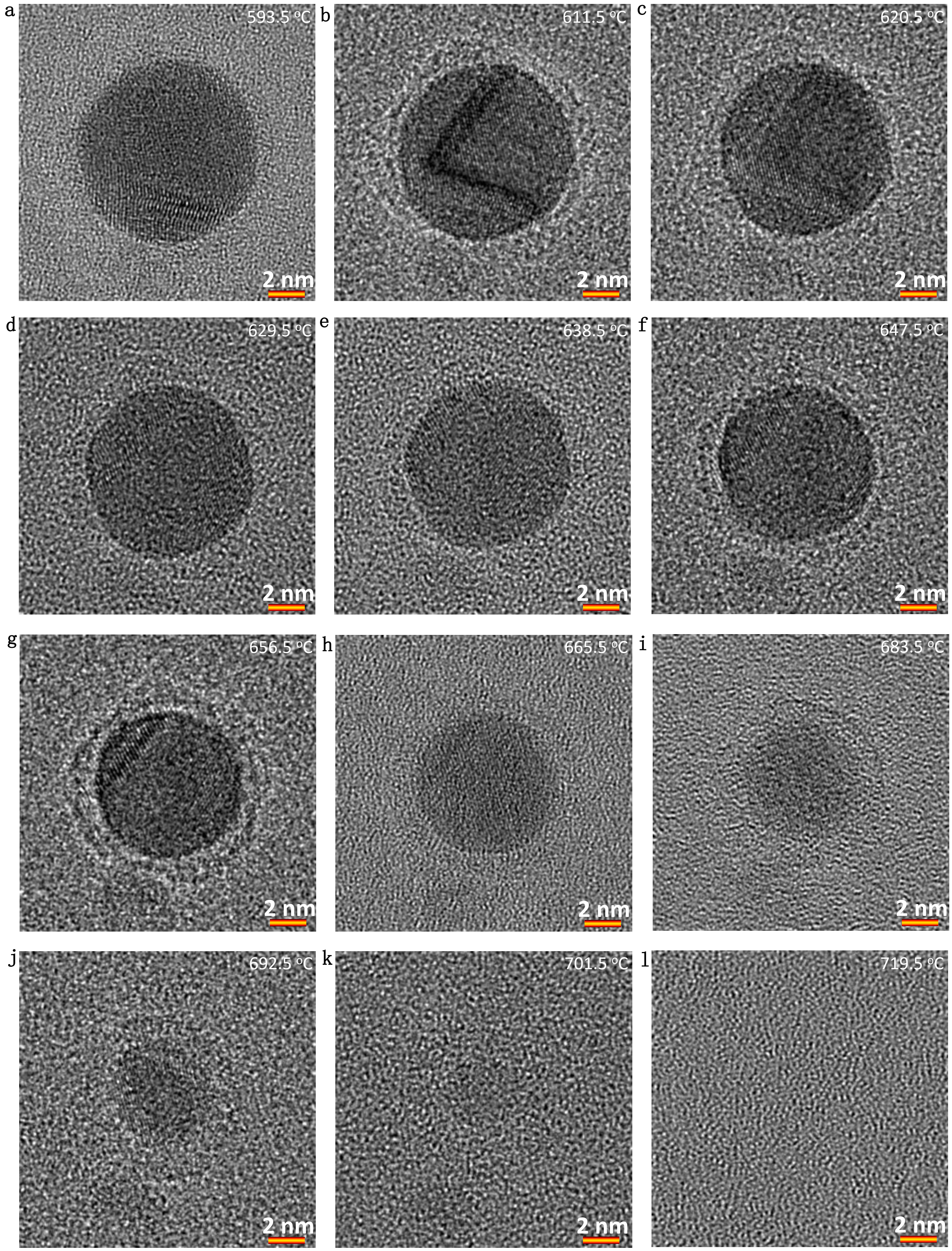

Supplementary Figure 7. The Enlarged sequential HRTEM images of the yellow box region in Figure S5 showing non-uniform sublimation.

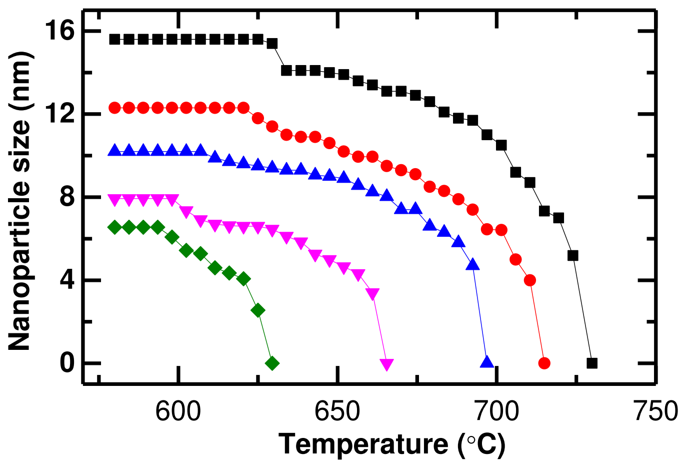

Supplementary Figure 8. Statistics of size-dependent sublimation temperature. Nanoparticle size as a function of temperature from ~570 ºC to ~730 ºC. The heating rate is ~ 0.5 ºC/s.

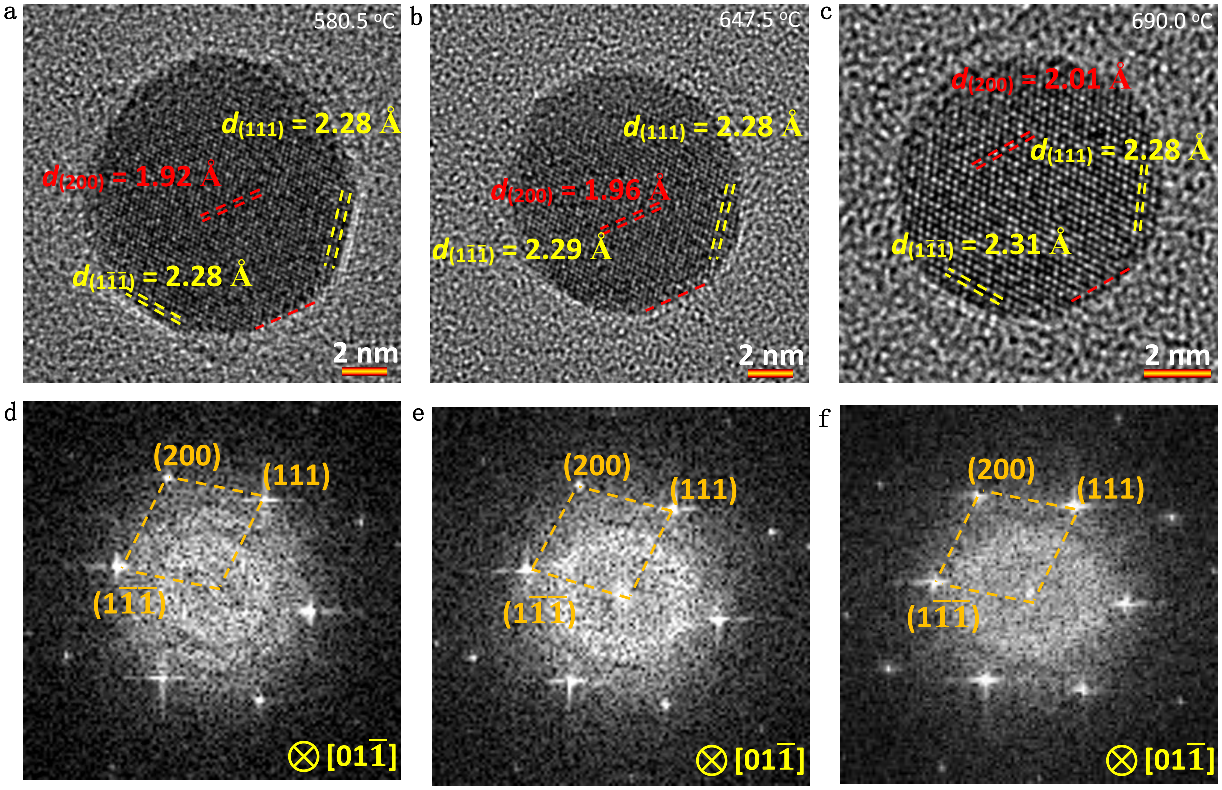

Supplementary Figure 9. Stable {111} and {200} planes during sublimation at different temperature of 580.5 ºC, 647.5 ºC and 690 ºC.

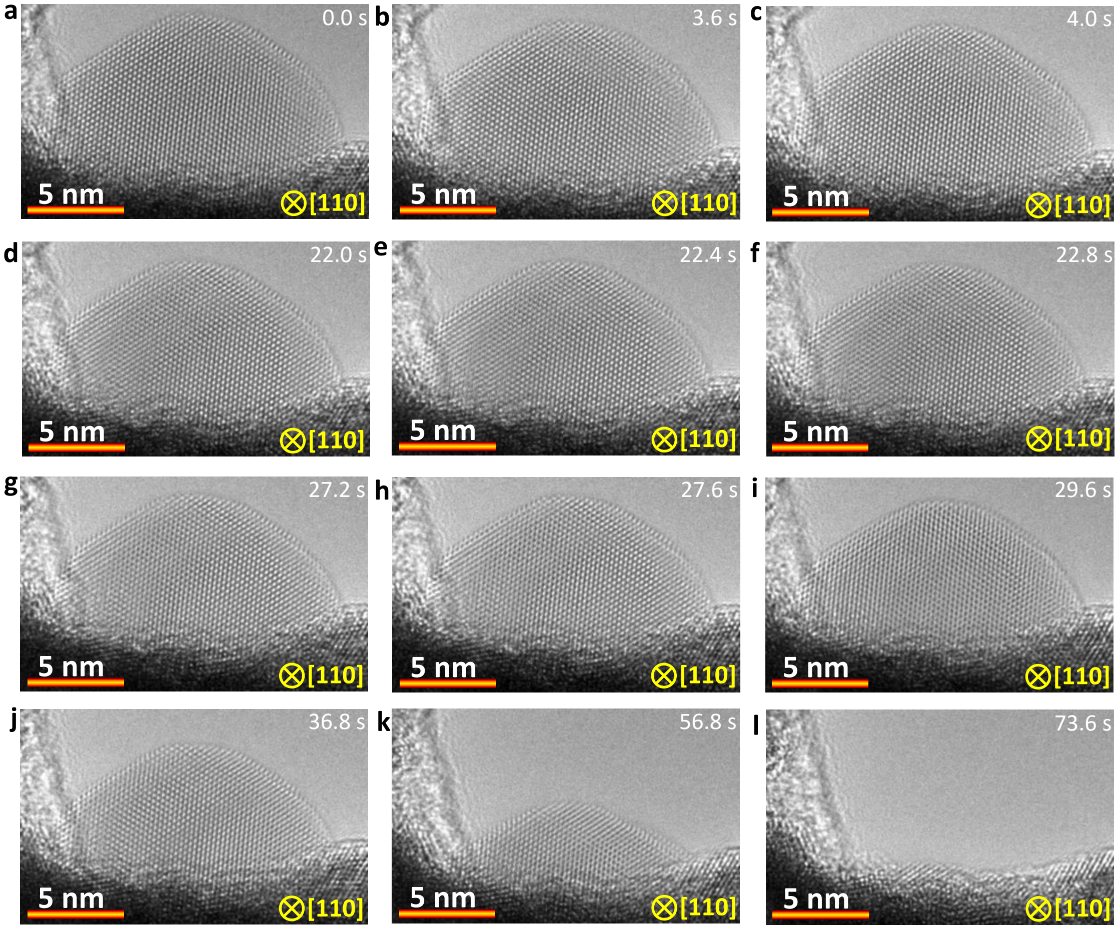

Supplementary Figure 10. Extra example showing low surface energy induced uniform sublimation. Time-sequential high resolution TEM images of uniform sublimation pathway of an Ag nanoparticle with a size of about 15 nm at 600 ºC.

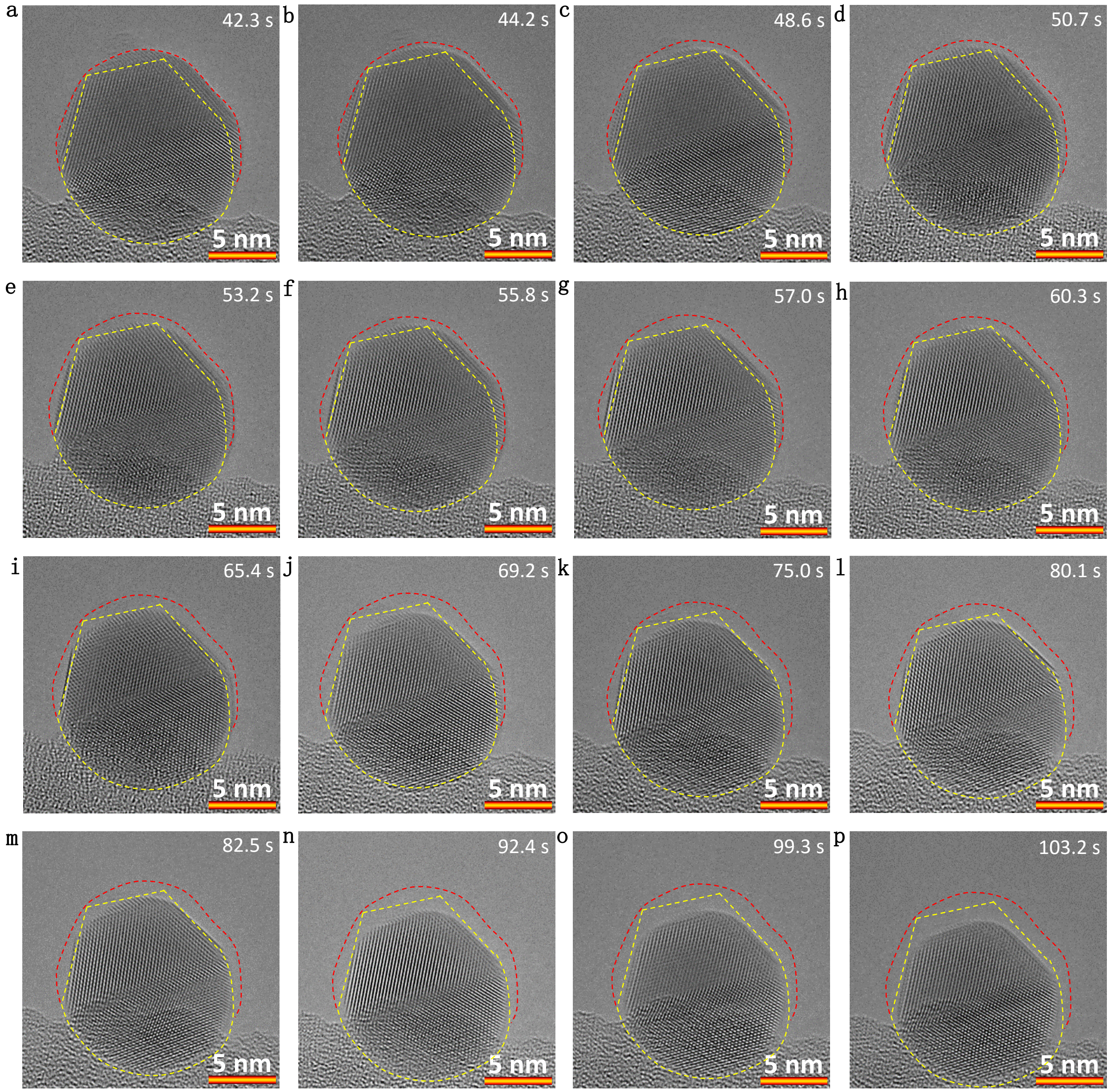

Supplementary Figure 11. Sequential HRTEM images showing the preferential sublimation in region II in Figure 3. Yellow and red curves clearly show the structural evolution of the nanocrystal during sublimation.

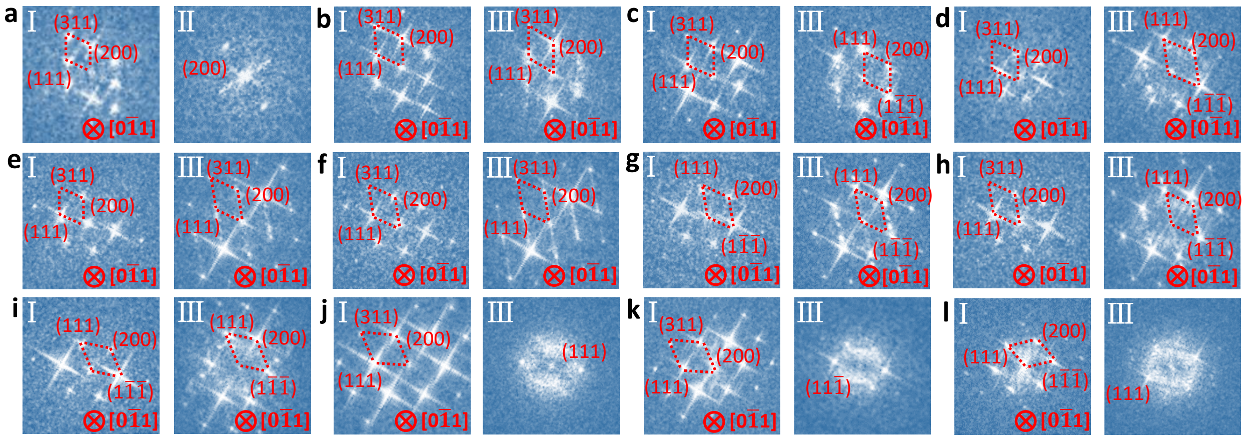

Supplementary Figure 12. Fast Fourier Transformation (FFT) analysis. Corresponding FFT pattern in marked region in Figure 3a-l.

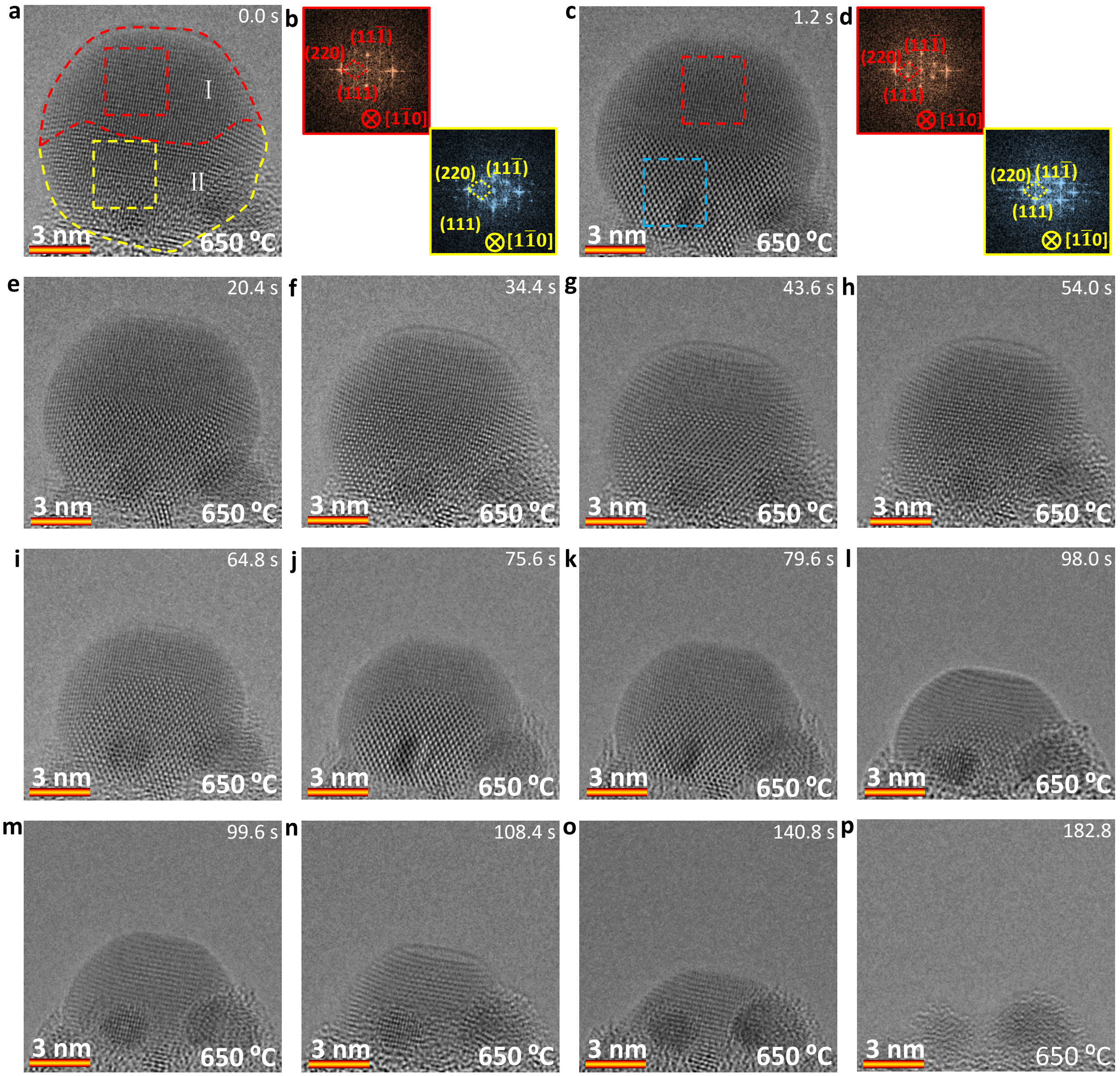

Supplementary Figure 13. Extra example showing high surface energy induced non-uniform sublimation. Time-sequential high resolution TEM images of non-uniform preferential sublimation in domain I (Figure 13 a) of a Ag nanoparticle with a size of about 11 nm at 650 ^{o}C. Figure S13b and S13d show the corresponding FFT analysis in the marked region in Figure S13a and S13c, respectively.

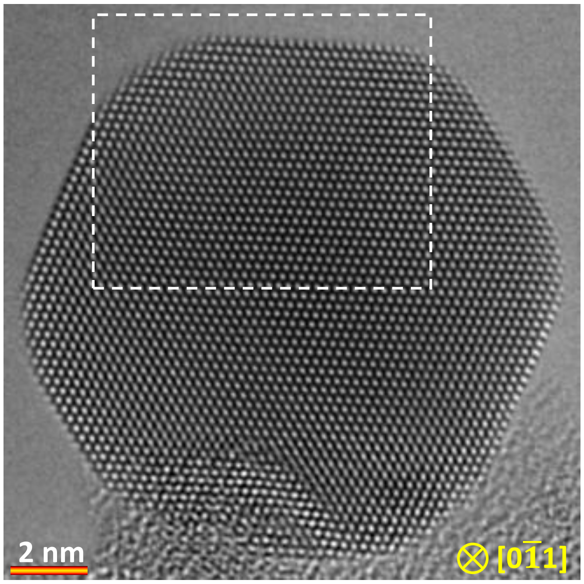

Supplementary Figure 14. The aberration-corrected HRTEM image for the strain tensor analyses in Video S1. The marked white box region in Video S1 are adapted for the strain tensor analyses during layer-by-layer sublimation of Ag nanocrystal.

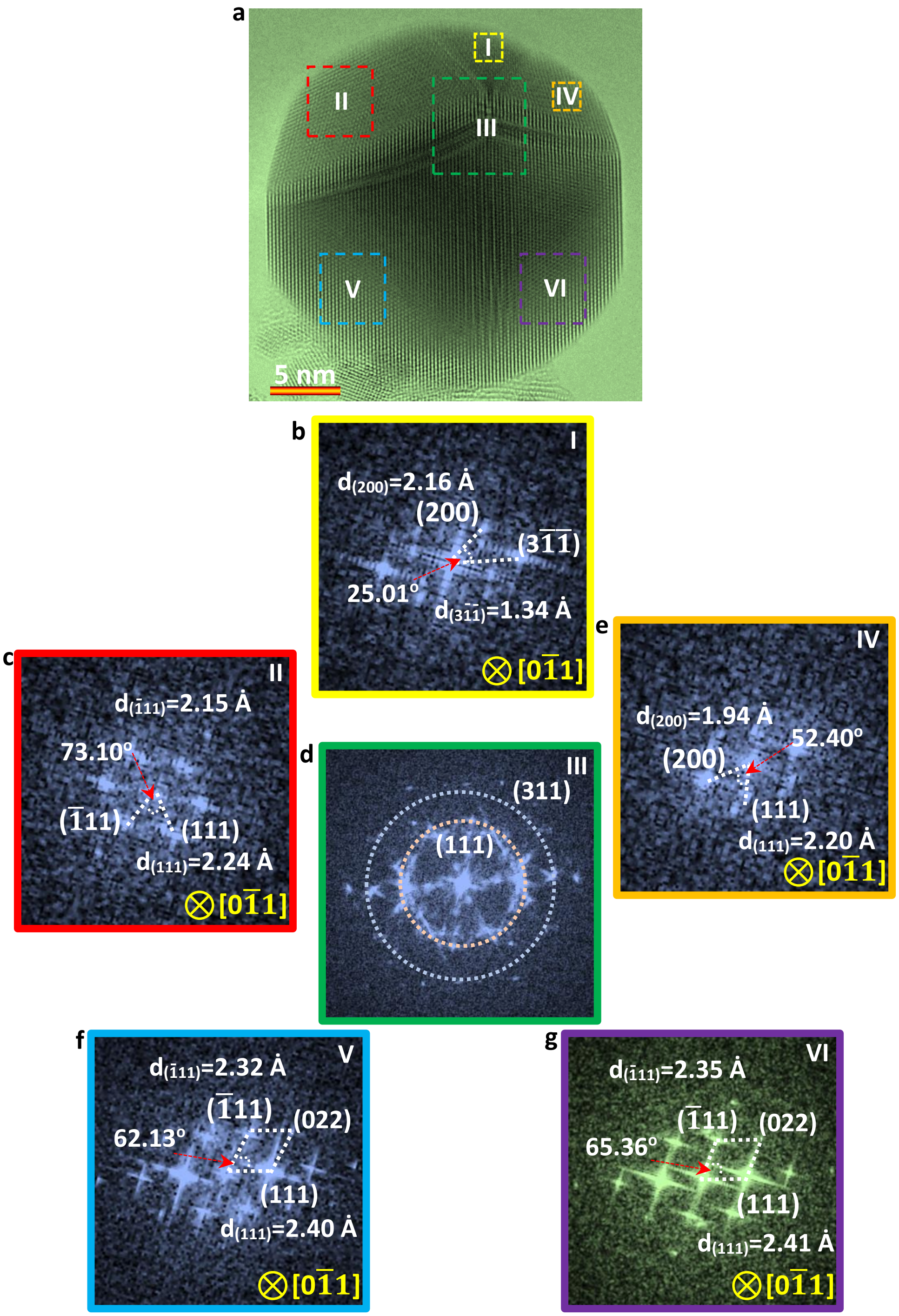

Supplementary Figure 15. FFT analysis in Figure 4a. The FFT analyses confirm the near 5-fold twin grain boundary structure in Figure 4a.

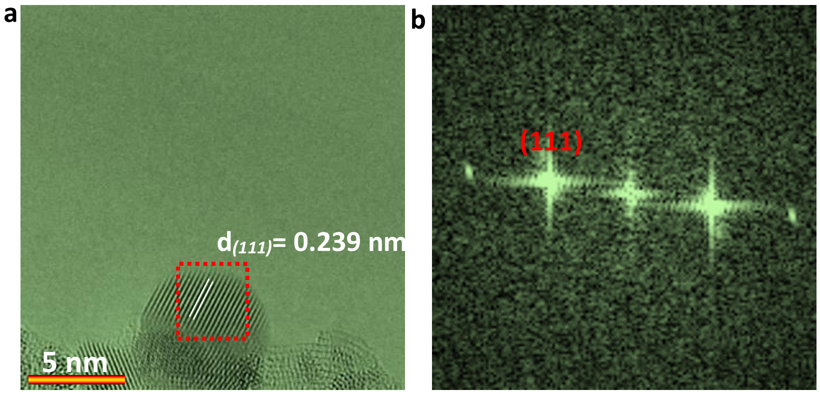

Supplementary Figure 16. FFT analysis in Figure 4k. The FFT analysis showing the structural rearrangement during sublimation in Figure 4k.

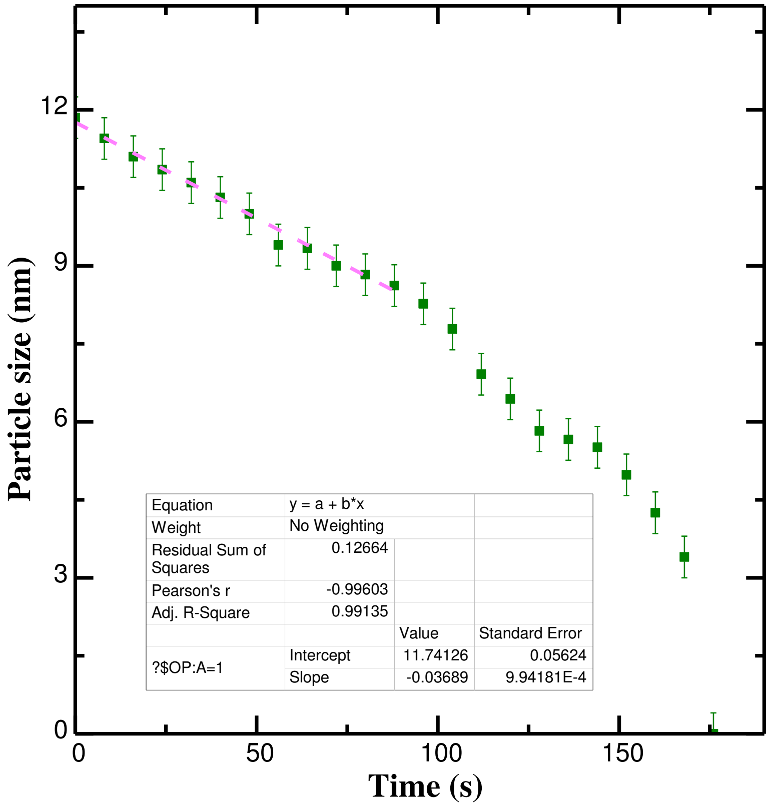

Supplementary Figure 17. Statistical analysis of sublimation dynamics at 650 ºC in supplementary Figure 13. The error bar is 0.4 nm.

Table S1. The error analyses of identification of zone axis in Figure S14. The experimental values are obtained based on the corresponding FFT analyses using Image J soft. The calculated values are obtained based the FCC structure of silver using Carine Crystallography. The error (E) = Experimental Value (plane 1)/ Calculated Value (plane 1) + Experimental Value (plane 2)/ Calculated Value (plane 2) + Experimental Value (Angle between the two planes)/ Calculated Value (Angle between the two planes).

Items / Figure	Plane spacing			Angle between two planes		Zone Axis	Error (E)
	Plane	Experimental Value (Å)	Calculated Value (Å)	Experimental Value (°)	Calculated Value (°)		
Figure S15b	(200)	2.16	2.04	25.01	25.24	[0$\bar{1}$1]	15.73%
	(3$\bar{1}\bar{1}$)	1.34	1.23				
Figure S15c	(111)	2.24	2.36	52.40	54.73	[0$\bar{1}$1]	16.40%
	($\bar{1}$11)	2.15	2.36				
Figure S15e	(200)	1.94	2.04	73.10	70.53	[0$\bar{1}$1]	15.32%
	(111)	2.20	2.36				
Figure S15f	(111)	2.40	2.36	30.12	35.26	[0$\bar{1}$1]	15.27%
	(022)	1.38	1.44	32.01	35.26		
	($\bar{1}$11)	2.32	2.36				
Figure S15g	(111)	2.41	2.36	31.15	35.26	[0$\bar{1}$1]	9.35%
	(022)	1.49	1.44	34.21	35.26		
	($\bar{1}$11)	2.35	2.36				

Video Information

Video S1. The uniform sublimation dynamic pathway in an Ag nanocrystal with low surface energy. The video was recorded at temperatures of 650 ^{o}C 4 times' normal speed. The electron dose rate is 8.0 $\times 10^3$ e/Å^{2}s.

Video S2. The non-uniform sublimation dynamic pathway in an Ag nanocrystal with high surface energy. The video was recorded at temperatures of 650 ^{o}C 8 times' normal speed. The electron dose rate is 8.0 $\times 10^3$ e/Å^{2}s.

Video S3. The defects structure induced non-uniform sublimation dynamic pathway in an Ag nanocrystal with 5-fold twin grain boundary. The video was recorded at temperatures of 650 ^{o}C and 10 times' normal speed. The electron dose rate is 8.0 $\times 10^3$ e/Å^{2}s.
